# Designing a Transparent and Fluorine Containing Hydrogel

**DOI:** 10.3390/gels7020043

**Published:** 2021-04-08

**Authors:** Paolo Ravarino, Demetra Giuri, Davide Faccio, Claudia Tomasini

**Affiliations:** Dipartimento di Chimica Giacomo Ciamician, Università di Bologna, Via Selmi, 2, 40126 Bologna, Italy; paolo.ravarino2@unibo.it (P.R.); demetra.giuri2@unibo.it (D.G.); davide.faccio2@unibo.it (D.F.)

**Keywords:** fluorine, hydrogel, pH change, rheology, thixotropy

## Abstract

Physical hydrogels are supramolecular materials obtained by self-assembly of small molecules called gelators. Aromatic amino acids and small peptides containing aromatic rings are good candidates as gelators due to their ability to form weak bonds as π-π interactions and hydrogen bonds between NH and CO of the peptide chain. In this paper we show our results in the preparation of a transparent hydrogel that was obtained by self-assembly of a fluorine-containing dipeptide that relies on the additional formation of halogen bonds due to the fluorine atoms contained in the dipeptide. We used Boc-D-F_2_Phe-L-Oxd-OH (F_2_Phe = 3,4-difluorophenylalainine; Oxd = 4-methyl-5-carboxy-oxazolidin-2-one) that formed a strong and transparent hydrogel in 0.5% *w*/*w* concentration at pH = 4.2. The formation of a hydrogel made of unnatural fluorinated amino acids may be of great interest in the evaluation of patients with parkinsonian syndromes and may be used for controlled release.

## 1. Introduction

Physical hydrogels are supramolecular materials obtained by self-assembly of small molecules called gelators that often contain one or more stereogenic centers. Aromatic amino acids and small peptides containing aromatic rings are good candidates as gelators due to their ability to form weak bonds as π-π interactions and hydrogen bonds between NH and CO of the peptide chain [1,2]. The presence of additional functional groups on the molecules may cause the formation of new weak bonds, which may help the formation of the supramolecular materials. Among these weak interactions we can list the tendency of halogenated amino acids to form halogen bonds or halogen-hydrogen bonds in the case of fluorine [3]. Li studied the gelation property of Fmoc-halogenated phenylalanines and found that the halogen substitution on the phenyl ring of phenylalanine could improve the gelation ability of the resulting compounds [4]. In particular, the efficient self-assembly and hydrogelation of Fmoc-protected pentafluorophenylalanine (Fmoc-F5-Phe) has been reported [1]. Suspensions of Fmoc-F5-Phe in water undergo rapid self-assembly to entangled fibrillar structures within minutes, giving rise to rigid supramolecular gels. Variation of the fluorinated aromatic side chain or N-terminal functionalization perturbs hydrogelation, implicating fluorine and π–π interactions as the primary determinants for molecular recognition and self-assembly. Moreover, the formation of a fluorinated hydrogel may be of great interest in the evaluation of patients with parkinsonian syndromes, since in nuclear medicine 6-fluoro-(18F)-L-Dopa (FDopa) positron emission tomography/computed tomography (PET/CT) is used to study the presynaptic dopaminergic pathway [5]. FDopa uptake reflects the integrity of dopaminergic pathway, the activity of L-Dopa-decarboxylase enzyme, and the storage capacity of dopamine [6].

The use of Fmoc-protected amino acids for the formation of hydrogels may have some severe limitations due to the procedure for hydrogel formation. In the classical method, the gelator is dissolved at a basic pH to help its dissolution. Upon cleavage of the Fmoc-group from a peptide chain, a highly reactive dibenzofulvalene is formed. While the toxicity of dibenzofulvalene coming from Fmoc-based peptides has not been determined directly, some studies have indicated that Fmoc-FF degradation products show some cytotoxicity [7]. Therefore, several groups are now developing new hydrogelators capped with groups different from Fmoc [8].

Our hydrogels may finally find an application as drug carriers. Many pharmaceutical agents are hydrophobic molecules, whose water solubility and bioavailability are limited. To improve these properties, drugs are often encapsulated in vehicles such as micelles or vesicles [9]. In addition, low molecular weight gels have been recently discovered for drug delivery [10]. The use of small peptides containing unnatural amino acids has been proved to be useful in drug delivery, as these chains are hardly recognized by enzymes [11,12]. In addition, a gel of a fluorine derivative of phenylalanine (Fmoc-F^5^-Phe-DAP, where DAP is 1,3-diaminopropane) has been recently tested as a vehicle for diclofenac in vivo [13]. The gel showed the ability to release the drug over two weeks.

## 2. Results and Discussion

**Figure 1:**
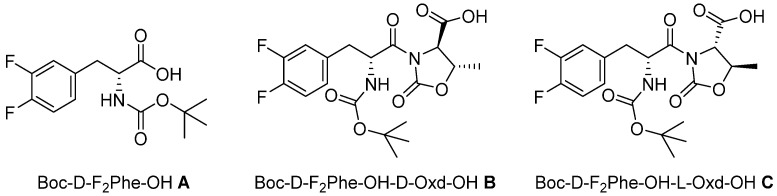
Chemical structure of gelators **A**, **B,** and **C**.

In this paper we show our results in the formation of supramolecular hydrogels, using the gelators **A, B,** and **C**, all containing the moiety D-F_2_Phe (3,4-difluorophenylalanine). As N protecting group, we used the *tert*-buxoxycarbonyl group, avoiding the use of the cytotoxic Fmoc (Figure 1) [7]. Among the other properties, we pursued the preparation of a transparent hydrogel, as it is highly desirable for biological applications.

The three gelators have been obtained as derivatives of D-F_2_Phe. The introduction of the Oxd moiety was done, taking into consideration our previous work, where we prepared supramolecular materials containing the moiety L-Phe-D-Oxd [14,15], or, more recently, the L-Tyr-D-Oxd [16,17] and L-Dopa-D-Oxd [18,19]. In particular, F_2_-Phe is similar to the structure of Dopa, where the hydroxyl groups have been replaced with fluorine atoms. In any case, the self-assembly of these molecules produced interesting supramolecular materials such as fibers and gels in several conditions. These properties are due to the presence of the imide group of the Oxd moiety, which introduces a constraint inside the molecule that usually favors the formation of intermolecular interactions [20]. Thus **A**, **B,** and **C** are promising gelators, due to the possible formation of several weak interactions, like hydrogen bonds, π-π stacking, and halogen-hydrogen bonds, from the fluorine atoms on the aromatic ring.

N-Boc derivative **A** may be purchased and was tested as it is. The two diastereomeric derivatives Boc-D-F_2_-Phe-D-Oxd-OH **B** and Boc-D-F_2_-Phe-L-Oxd-OH **C** were obtained by peptide coupling of **A** with D-Oxd-OBn or L-Oxd-OBn, both synthesized following the procedure reported by Falb [21], in solution, followed by the cleavage of the benzyl group by hydrogenolysis (Scheme S1).

All the hydrogels were prepared using the pH change method with concentrations of 0.5%, 1.0%, and 2.0% *w*/*w* concentration, after experiments to find the minimum gelation concentration (MGC, Figure S1, and Figure S2). The gelator was dissolved in water in concentrations ranging between 0.5% and 2.0% *w*/*w*, then 1M NaOH (1.3 e.) was added, and the mixture was sonicated for 30 min to dissolve the gelator. Thereafter, GdL (δ-gluctonolactone) (1.4 equivalent) was added to the mixture and was left to rest for several hours. The details and the results are summarized in Table 1.

**Figure 2:**
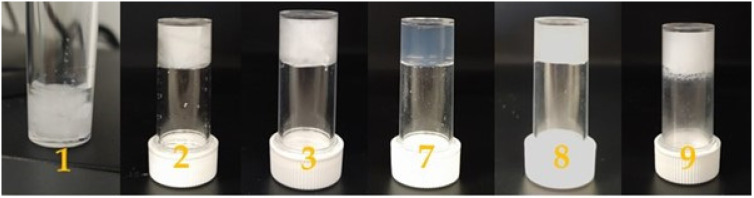
Photographs of hydrogels **1**–**3** and **7**–**9**.

**Figure 3:**
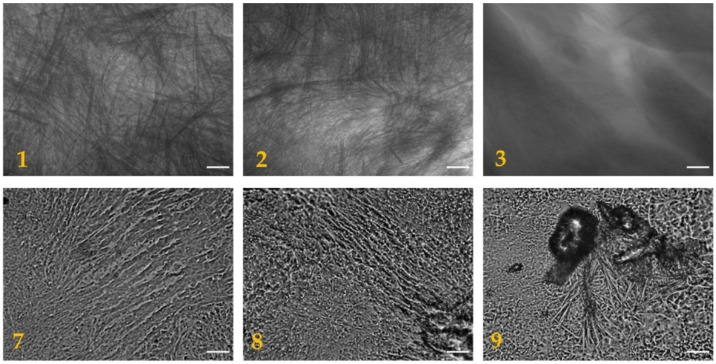
Optical microscope images of dried hydrogels **1**–**3** (scale bar: 100 µm) and **7**–**9** (scale bar: 25 µm).

**Figure 4:**
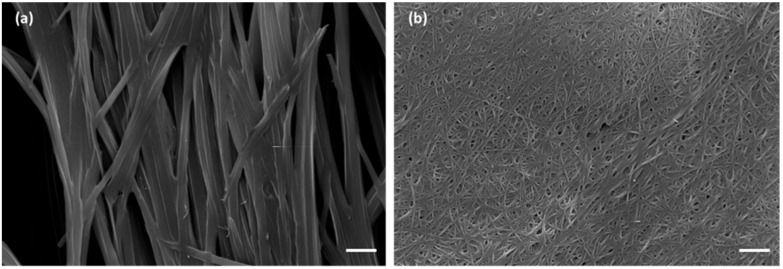
Scanning electron microscope (SEM) images of dried hydrogels (**a**) **1** and (**b**) **4** (scale bar: 5 µm).

Both compounds **A** and **C** promote the formation of hydrogels as shown in Figure 2. The morphology of the dried hydrogels was analyzed through optical microscopy, as shown in Figure 3, and through scanning electron microscopy (SEM), as shown in Figure 4. The pictures show the presence of thick micrometric fibers in dried hydrogels **1**–**3** and of a dense network in dried hydrogels **7**–**9**. Similar results were obtained with the analysis of the corresponding hydrogels (Figures S3 and S4).

**Figure 5:**
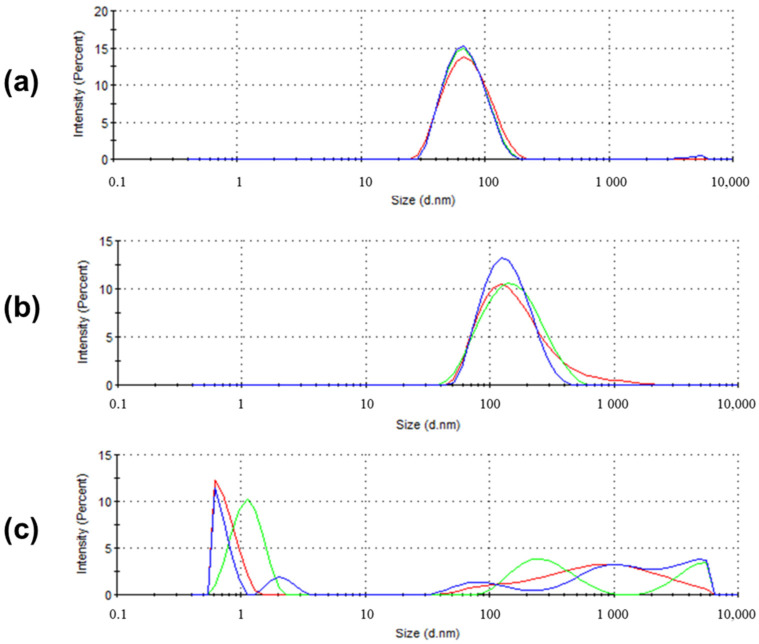
Size distribution by intensity of the particles from solutions of gelator **B** at different concentration: (**a**) 0.5 *w*/*w*; (**b**) 1.0% *w*/*w*; (**c**) 2.0% *w*/*w*.

In contrast, Boc-D-F_2_Phe-D-Oxd-OH **B** did not form a gel under any condition. To test if an aggregation occurred at the micrometric level, three solutions of **B** of different concentrations (0.5%, 1.0%, 2.0% *w/w*) were analysed by dynamic light scattering (DLS), after the procedure for hydrogel formation failed. We could see the formation of nanoaggregations of an average size of 65.63 nm (PdI = 0.148) for 0.5% and 130.1 nm (PdI = 0.254) for 1.0% concentration. For the solution of 2.0% concentration, some other aggregates of different size were detected, in agreement with the higher concentration used (Figure 5 and Figure S5).

The different behavior between the two diastereoisomers **B** and **C** is not surprising, as the absolute configuration is always crucial for the selection of a good gelator, which must have the tendency to form β-sheet structures, rather than turns, as we previously verified for oligomers of the L-Phe-D-Oxd and L-Phe-L-Oxd series [15,22]. We demonstrated in the past that the stereoisomers Boc-L-Phe-D-Oxd-OBn and Boc-L-Phe-L-Oxd-OBn adopt different conformations that are responsible for different properties; Boc-L-Phe-D-Oxd-OBn is a solid that spontaneously forms fibers consisting of infinite linear chains, in which the parallel dipeptide units are connected only by a single hydrogen bond [14]. In contrast, Boc-L-Phe-L-Oxd-OBn is a waxy solid, unable to form fibers. Detailed DFT computational analysis has been conducted for Boc-L-Phe-L-Oxd-OBn, to define the most populated conformers that have been identified. They exhibit backbone dihedral angles in the same range as those of a PPII (polyproline II) geometry [22]. Replacing Phe with F_2_Phe, the same properties were detected, suggesting that the molecules adopt the same preferred conformations already registered for the analogs that do not contain fluorine.

**Figure 6:**
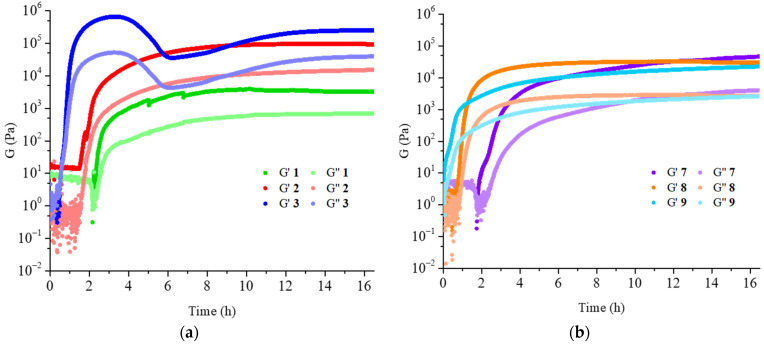
Time sweep of hydrogels (**a**) **1**–**3** and (**b**) **7**–**9**.

The rheological analysis of compounds **1**–**3** and **7**–**9** was performed. Time sweep accounts for the time needed to form the hydrogels (Figure 6). Although with different speed, the gels were always completely formed after 16 h.

The results obtained for hydrogels **1***–***3** were perfectly reasonable, with both storage modulus and loss modulus that increased with the increase of the gelator concentration and were supported by the analysis of the amplitude sweeps (Table 2 and Figures S6–S8), confirming the good performances of the hydrogels. Hydrogel **1** was a partial gel, as some water was released, but the gelated part showed good rheological properties.

In contrast, the results obtained for hydrogels **7**–**9** were surprising, as hydrogel **7** seems the strongest, even though it contained the lowest gelator concentration among the three samples. After careful analysis of the gelator and of the freezing conditions, the LC-MS analysis of both molecules **B** and **C** showed partial hydrolysis after dissolution in NaOH aqueous solution (Figure S9). For this reason, we suggest that the purity of the gelator after the dissolution should be always checked. 

To avoid these problems, we prepared a 0.5% *w*/*w* solution of gelators **B** and **C**, following the method described above and replacing the 1M NaOH solution with 0.1 M phosphate buffer solution at pH 7.4. This new method for the gelator dissolution was of great help when dissolving pH-sensitive molecules as gelators **B** and **C**. Gelator **B** did not form any gel, while gelator **C** formed a transparent hydrogel, named **10**. The low concentration of 0.5% was preferred as the hydrogel obtained with gelator **C** under these conditions was strong, transparent, and reproducible.

**Figure 7:**
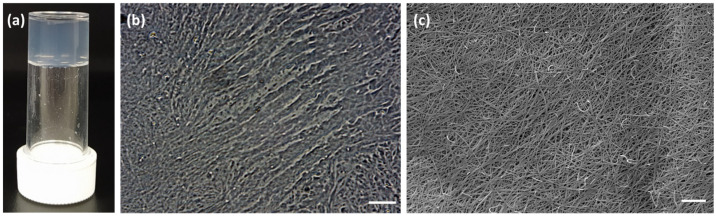
(**a**) Photograph of hydrogel **10** and pictures of fibrillar assemblies formed by dried hydrogel **10** obtained with (**b**) optical microscope (scale bar: 25 µm) and (**c**) scanning electron microscope (scale bar: 5 µm).

Therefore, we focused our attention on the formation of hydrogel **10**, obtained using gelator **C** in 0.5% *w*/*w* concentration. The gelator was dissolved in a solution of water with 1.3 equivalent of 0.1M phosphate buffer, using 1.4 equivalent of GdL as the trigger. This hydrogel was highly interesting, because it was transparent, strong, and had a final pH of 4.2 (Figure 7 and Figure S11). These results make this hydrogel an intriguing candidate for several applications. Transparent hydrogels incorporating a photocatalyst have been used for pollutant degradation [23]. Transparency is also highly desirable for other purposes, like cell culture, as it allows observation of the single cells [24], topical vehicles for pharmaceuticals, and cosmetics preparations [25,26,27]. Hypothetically, this gelator could be used as injectable solutions that form gels in situ postinjection, since the solutions are prepared in phosphate buffer solution (PBS) and the gel can only be formed in presence of an acidic pH. This possibility further increases the field of applicability of the system in drug delivery or imaging, especially for tumor targeting [28].

Finally, if isotopically labeled with ^18^F, these hydrogels may also find application as radiotracers in positron emission tomography/computed tomography (PET/CT) [5,29]. 

**Figure 8:**
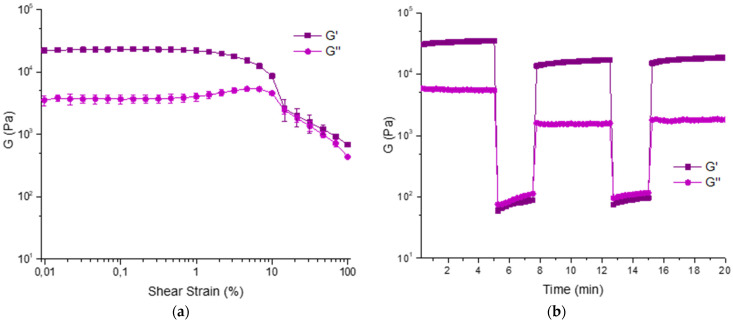
Rheological analysis of hydrogel 10 (**a**), amplitude sweep, and (**b**) step strain experiment.

We analyzed its rheological properties by time sweep, amplitude sweep, and thixotropic analysis. The formation of the gel is very fast as it is completely formed after 4 h (Figure S10). The amplitude sweep was repeated in triplicate and accounts for good properties as G′ = 22.99 ± 1.35 KPa and G″ = 3.71 ± 0.60 KPa (γ = 0.046%). The thixotropy analysis confirms these results and show that the hydrogel has good properties of recovery after a shear stress (Figure 8).

The driving forces that lead to the hydrogelation of gelators **A** and **C** may include the formation of halogen-hydrogen bonds, as previously suggested. To prove the presence of this additional weak interaction, ^19^F-NMR spectra of the two gelators were done before and after the gelation process (Figure S12). In any case a weak deshielding effect of the fluorine signals was observed, being more significative only for one signal of gelator **C** (0.195 ppm, corresponding to a shift of 78 Hz). Even though this variation in the chemical shift was low, it agreed with the small energy associated with the halogen-hydrogen bond, ranging between 0.5 and 1.6 kcal/mol [3].

## 3. Conclusions

In this paper we have demonstrated that we can prepare hydrogels with very good mechanical properties, avoiding the use of an aromatic protecting group on the N-terminal. Hydrogels have been obtained using mono- and dipeptides containing F_2_Phe that allow the formation of halogen-hydrogen bonds, in addition to π-π stacking and NH-OC bonds. In particular, the dipeptide Boc-D-F_2_Phe-L-Oxd-OH **C** forms a strong and transparent hydrogel at 0.5% *w*/*w* concentration and pH = 4.2. This hydrogel is a good candidate for several applications, such as the evaluation of patients with parkinsonian syndromes or controlled release.

Finally, a new method for the gelator dissolution has been tuned. The use of a bland basic environment strongly limits possible side reactions, allowing pH-sensitive gelators to be fully dissolved without being chemically modified. 

## 4. Materials and Methods

### 4.1. General Remarks for the Synthetic Procedure

Solvents were dried by distillation before use. All reactions were carried out in dried glassware. The melting points of the compounds were determined in open capillaries and are uncorrected. High quality infrared spectra (64 scans) were obtained at 2 cm^−1^ resolution with an FT-IR Bruker (Billerica, MA, USA) Alpha System spectrometer. All spectra were obtained in 3 mM solutions in CH_2_Cl_2_. All compounds were dried in vacuo and all the sample preparations were performed in a nitrogen atmosphere. NMR spectra were recorded with a Varian (Palo Alto, CA, USA) Inova 400 spectrometer at 400 MHz (^1^H NMR), at 100 MHz (^13^C NMR), and at 376.5 MHz (^19^F NMR). Chemical shifts are reported in δ values relative to the solvent peak. HPLC-MS was used to check the purity of compounds.

### 4.2. Preparation of Boc-D-F_2_-Phe-D-Oxd-OH B

Boc-D-F_2_-Phe-OH (500 mg, 1.66 mmol) was dissolved in 20 mL of ACN and then HBTU (693 mg, 1.83 mmol) was added. The mixture was stirred at room temperature for 10 min. A solution containing D-Oxd-OBn (390 mg, 1.66 mmol) and DIEA (0.9 mL, 5.31 mmol) in ACN (10 mL) was then added dropwise to the first one. The mixture was stirred for 6 h, then the solvent was removed under reduced pressure and replaced with ethyl acetate (40 mL). The organic mixture was washed with H_2_O (10 mL), 1N aqueous HCl (10 mL), and brine (10 mL), then it was dried over Na_2_SO_4_ and the solvent evaporated under vacuum. The solid obtained was finally purified through flash chromatography (cyclohexane:ethyl acetate 4:1). Boc-D-F_2_-Phe-D-Oxd-OBn was obtained as a white solid and directly hydrogenolysed. 

In a flask containing Boc-D-F_2_-Phe-D-Oxd-OBn (700 mg, 1.49 mmol) and methanol (50 mL), Pd/C 10% *w/w* (70 mg) was added to the solution. The air left in the flask was removed through a water pump, then the mixture was posed under hydrogen atmosphere and stirred for 2 h until the reaction was complete, then it was filtered on a Celite pad. The solution was evaporated under reduced pressure and the product was obtained as a white solid in 89% overall yield. M.p. = 112°C (dec.); [α]^25^_D_ +3.0° (c = 0.5 in EtOAc); IR (ATR-IR): ν 3276, 2978, 2935, 1785, 1716, 1644, 1610, 1518 cm^−1^; ^1^H NMR (CD_3_OD, 400 MHz): δ 1.33 (9H, s, CH_3_ t-Bu), 1.55 (3H, d, J = 6.4 Hz, CH_3_ Oxd), 2.63 (1H, dd, J = 11.2, 13.6 Hz, CH benzyl), 3.26 (1H, m, CH benzyl), 4.58 (1H, d, J = 4.0 Hz, CαH Oxd), 4.74 (1H, m, CβH Oxd), 5.46 (1H, d, J = 8.8 Hz, CαH F_2_-Phe), 5.57 (1H, d, J = 9.2 Hz, NH-Boc), 7.20 (3H, m, CH aromatic); ^13^C (CD_3_OD, 100 MHz): δ 19.79, 27.15, 36.16, 54.72, 61.66, 74.74, 79.31, 116.44, 117.89, 125.58, 134.79, 148.25, 150.69, 152.53, 156.51, 170.04, 172.26; ^19^F (CD_3_OD, 376.5 MHz): δ −143.97, −141.23.

### 4.3. Preparation of Boc-D-F_2_-Phe-L-Oxd-OH C

The product was prepared following the synthetic procedure described for the preparation of B and replacing D-Oxd-OBn with L-Oxd-OBn, overall yield 88%. M.p. = 142 °C (dec.); [α]^25^_D_ −38.0° (c = 0.5 in EtOAc); IR (ATR-IR): ν 3358, 2982, 2933, 1779, 1722, 1683, 1610, 1518 cm^−1^; ^1^H NMR (CD_3_OD, 400 MHz): δ 1.31 (9H, s, CH_3_ t-Bu), 1.50 (3H, d, J = 6.4Hz, CH_3_ Oxd), 2.66 (1H, dd, J = 15.6, 10.4 Hz, CH benzyl), 3.13 (1H, m, CH benzyl), 4.43 (1H, d, J = 4.0 Hz, CαH Oxd), 4.73 (2H, dq, J = 6.4, 4.0 Hz, CβH Oxd), 5.58 (1H, dd, J = 10.0, 3.6 Hz, CαH F_2_-Phe), 7.16 (3H, m, CH aromatic); ^13^C (CD_3_OD, 100 MHz): δ 19.75, 27.14, 37.12, 54.39, 62.10, 74.76, 79.08, 116.45, 117.95, 125.58, 134.79, 148.25, 150.69, 152.53, 156.51, 170.04, 172.26; ^19^F (CD_3_OD, 376.5 MHz): δ -143.98, -141.26.

### 4.4. Rheological Analysis

All rheological measurements were performed using an Anton Paar (Graz, Austria) MCR102 rheometer. A vane and cup measuring system was used, setting a gap of 2.1 mm. The gels were prepared as described and tested directly in the Thermo Fisher Scientific (Waltham, MA, USA) Sterilin cup, which fits in the rheometer. Time sweep experiments were performed at 23 °C (controlled by an integrated Peltier system) using a constant shear strain (γ) of 0.5% and a constant angular frequency (ω) of 10 rad/s, collecting 1 point every 20 s. Oscillatory amplitude sweep experiments (γ: 0.01−100%) were also performed at 23 °C using a constant angular frequency of 10 rad/s. Step strain experiments were performed on hydrogels, subjecting the sample to consecutive deformation and recovery steps. The recovery step was performed by keeping the sample at a constant strain γ = 0.03%, i.e., within the LVE region, for a period of 400 s. The deformation step was performed by applying to the gel a constant strain of γ = 100%, i.e., above the LVE region of the sample, for a period of 300 s. The cycles were performed at a fixed frequency of ω = 10 rad s^−1^ and repeated three times.

### 4.5. Optical Microscope Images

The optical microscope images were recorded using a Nikon (Minato, Japan) 13 ECLIPSE Ti2 Inverted Research Microscope with a 10× or 40× magnifier. A piece of the gel sample prepared in the Sterilin cups was cut using a bistoury and analysed both while wet and after complete drying.

### 4.6. Scanning Electron Microscopy

Scanning electron micrographs of the samples were recorded using a Hitachi 6400 field emission gun scanning electron microscope operating at 15 kV (Hitachi, Chiyoda, Tokyo, Japan).

## Figures and Tables

**Table 1:** Results for the formation of hydrogels from **A**, **B** and **C**, using the pH change method.

Sample	Compound	Concentration (% *w*/*w*)	Result
**1**	**A**	0.5	partial gel
**2**	**A**	1	opaque gel
**3**	**A**	2	opaque gel
**4**	**B**	0.5	solution
**5**	**B**	1	opaque solution
**6**	**B**	2	opaque solution
**7**	**C**	0.5	transparent gel
**8**	**C**	1	opaque gel
**9**	**C**	2	white gel

**Table 2:** G’ and G’’ moduli from amplitude sweep (γ = 0.046%) for compounds **1**–**3**.

Sample	G′ (kPa)	G″ (kPa)
**1**	17.50 ± 5.11	1.45 ± 0.54
**2**	92.67 ± 41.46	6.20 ± 3.56
**3**	467.66 ± 60.89	29.97 ± 6.40

## Data Availability

The data presented in this study are available on request from the corresponding author.

## Supplementary Materials

The following are available online at https://www.mdpi.com/article/10.3390/gels7020043/s1, Scheme S1: Synthesis of Boc-D-F2-Phe-D-Oxd-OH **B** and Boc-D-F2-Phe-L-Oxd-OH C, with yields after flash chromatography. Figure S1. Analysis of the minimum gelation concentration (MGC) need to form hydrogels from **A**. Figure S2. Analysis of the minimum gelation concentration (MGC) need to form hydrogels from C. Figure S3. Hydrogel images of **1**, **2** and **3** obtained with an optic microscope with a 10x magnification. Figure S4. Hydrogel images of **7**, **8** and **9** obtained with an optic microscope with a 40x magnification. Figure S5. DLS correlation coefficient, number and volume analysis of particles after filtration: (a) solution **4**; (b) solution **5**; (c) solution **6**. Figure S6. Amplitude sweep analysis of hydrogel **1**. Figure S7. Amplitude sweep analysis of hydrogel **2**. Figure S8. Amplitude sweep analysis of hydrogel **3**. Figure S9. HPLC-MS analysis of gelators **B** and **C** before and after the addition of GdL. Figure S10. Time sweep analysis of hydrogel **10**. Figure S11. Absorbance spectrum of hydrogel **10**. Figure S12: 19F-NMR spectra registered in D2O of gelators **A** and **C** before and after the gelation process.
