# Supplementary material for: Designing a Transparent and Fluorine Containing Hydrogel
Source: Gels. 2021 Apr 8;7(2):43. doi: 10.3390/gels7020043 (PMC8167729; doi:10.3390/gels7020043)
Supplement: Supplementary file 1 [file gels-07-00043-s001.pdf]

Supplementary Materials

Contents

**Scheme S1.** Preparation of the gelators **B** and **C**	Page S2
NMR and IR spectra of compounds **B** and **C**	Pages S3-S8
**Figure S1.** Analysis of the minimum gelation concentration (MGC) need to form hydrogels from **A**.	Page S9
**Figure S2.** Analysis of the minimum gelation concentration (MGC) need to form hydrogels from **C**.	Page S9
**Figure S3.** Hydrogel images of **1, 2** and **3** obtained with an optic microscope	Page S10
**Figure S4.** Hydrogel images of **7, 8** and **9** obtained with an optic microscope	Page S10
**Figure S5.** DLS correlation coefficient, number and volume analysis of **4**, **5** and **6**	Page S11
**Figure S6.** Amplitude sweep analysis of hydrogel **1**	Page S12
**Figure S7.** Amplitude sweep analysis of hydrogel **2**	Page S12
**Figure S8.** Amplitude sweep analysis of hydrogel **3**	Page S13
**Figure S9.** HPLC-MS analysis of gelators **B** and **C** after gelation process, showing different degrees of hydrolysis	Page S14
**Figure S10.** Time sweep analysis of hydrogel **10**	Page S15
**Figure S11.** Absorbance spectrum of hydrogel **10**	Page S16
**Figure S12.** ^{19}F-NMR spectra of gelators **A** and **C** before and after the gelation process	Page S17

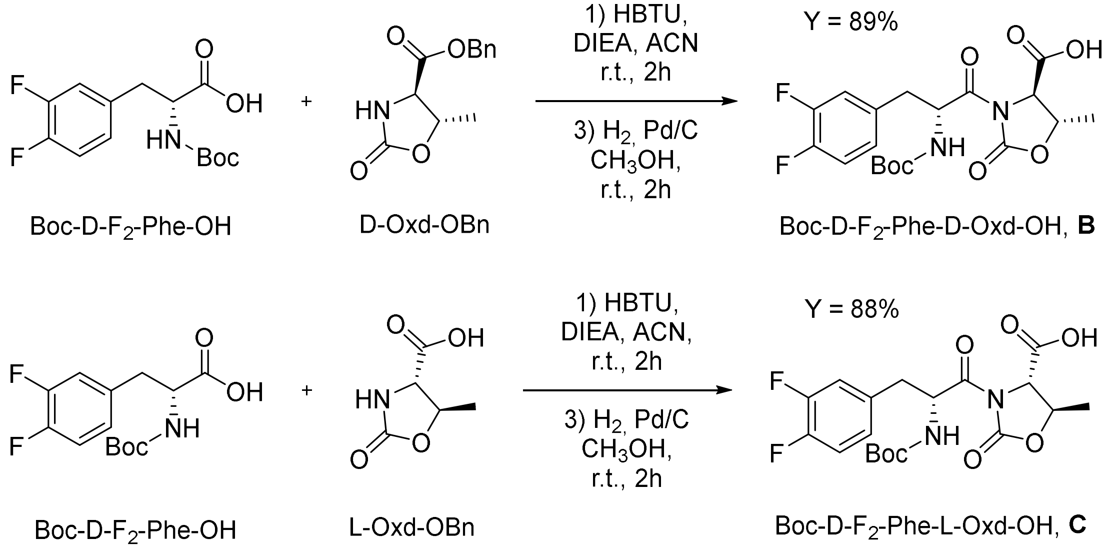

Scheme 1. Synthesis of Boc-D-F$_2$-Phe-D-Oxd-OH **B** and Boc-D-F$_2$-Phe-L-Oxd-OH **C**, with yields after flash chromatography.

^{1}H NMR spectrum of Boc-D-F_2-Phe-Oxd-OH **B** in CD_3OD

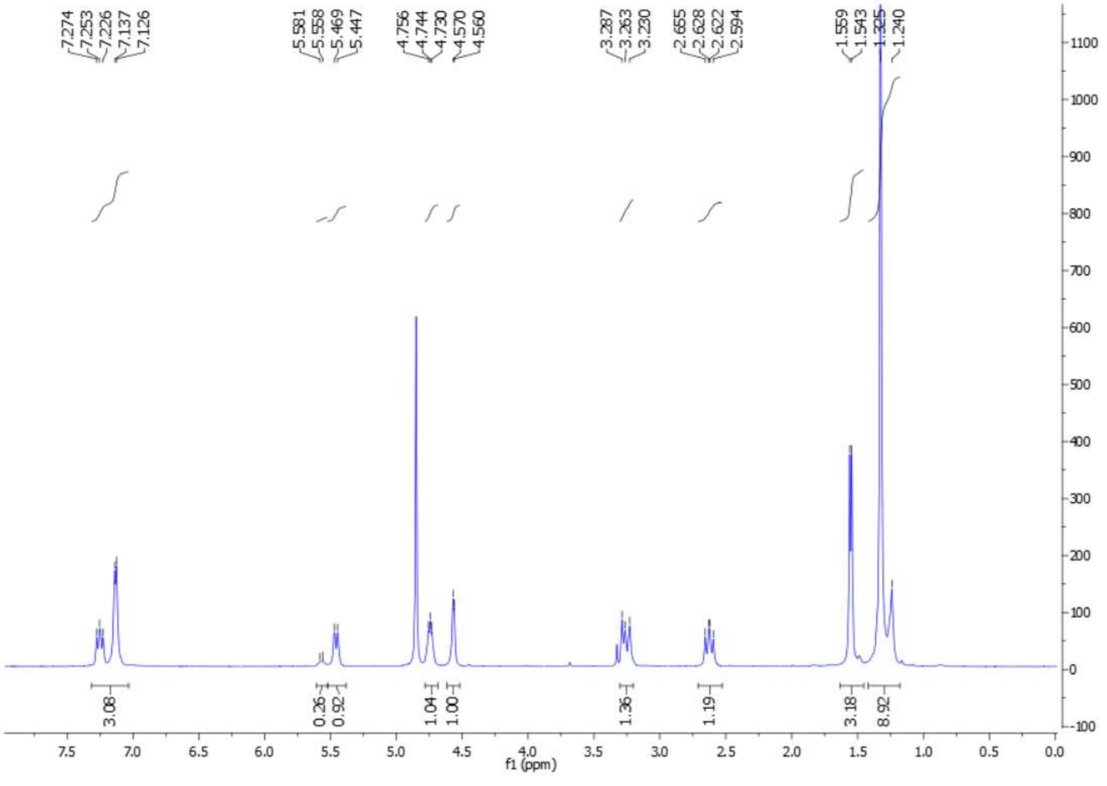

COSY spectrum of Boc-D-F_2-Phe-D-Oxd-OH **B** in $CDCl_3$

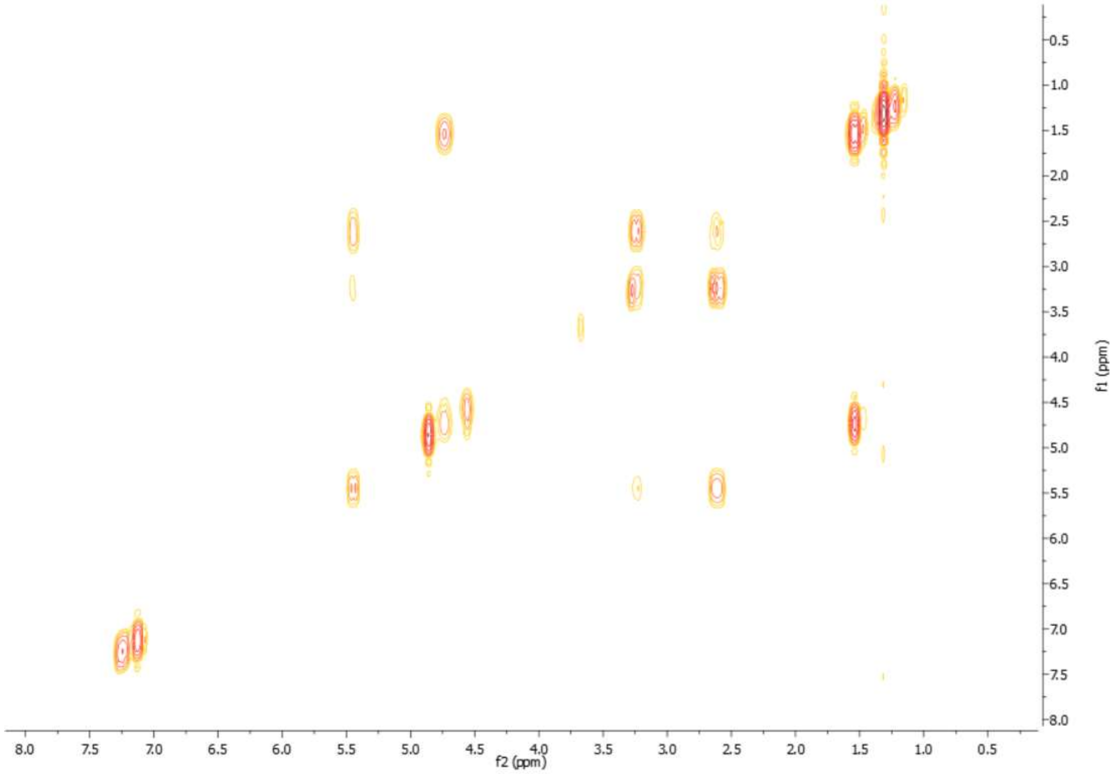

^{13}C NMR spectrum of Boc-D-F$_2$-Phe-D-Oxd-OH **B** in CD_3OD

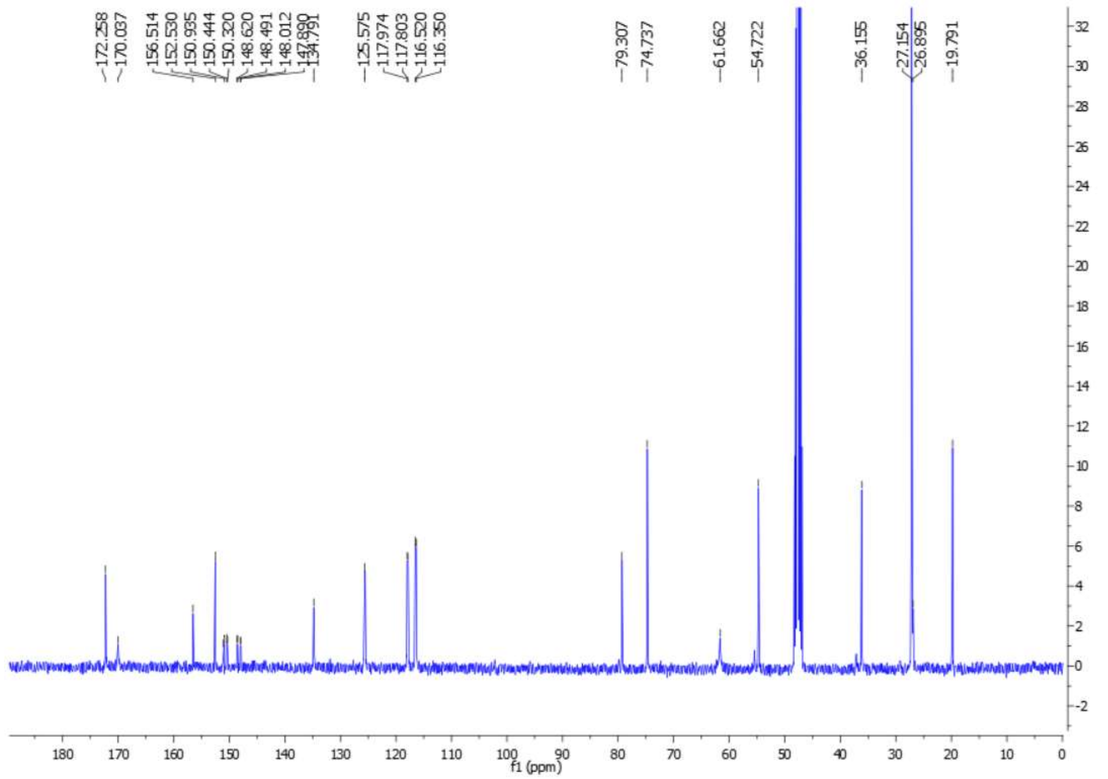

^{19}F NMR spectrum of Boc-D-F$_2$-Phe-D-Oxd-OH **B** in $CDCl_3$

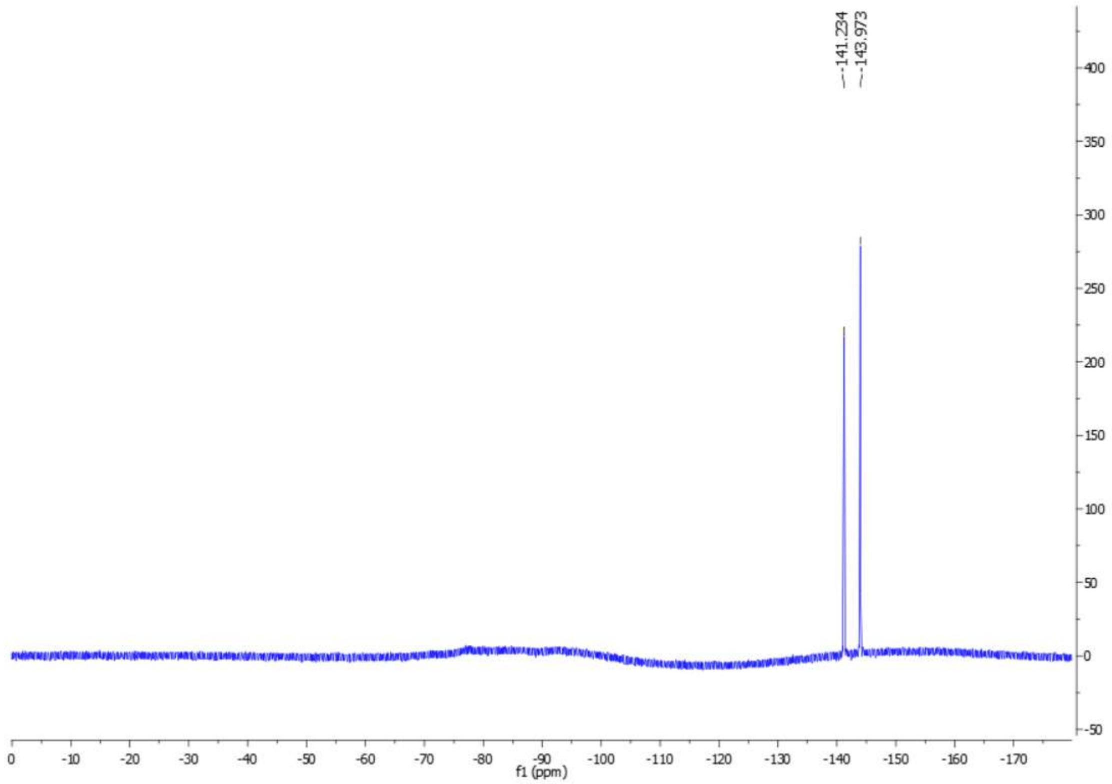

IR-ATR spectrum of Boc-D-F$_2$-Phe-D-Oxd-OH **B**

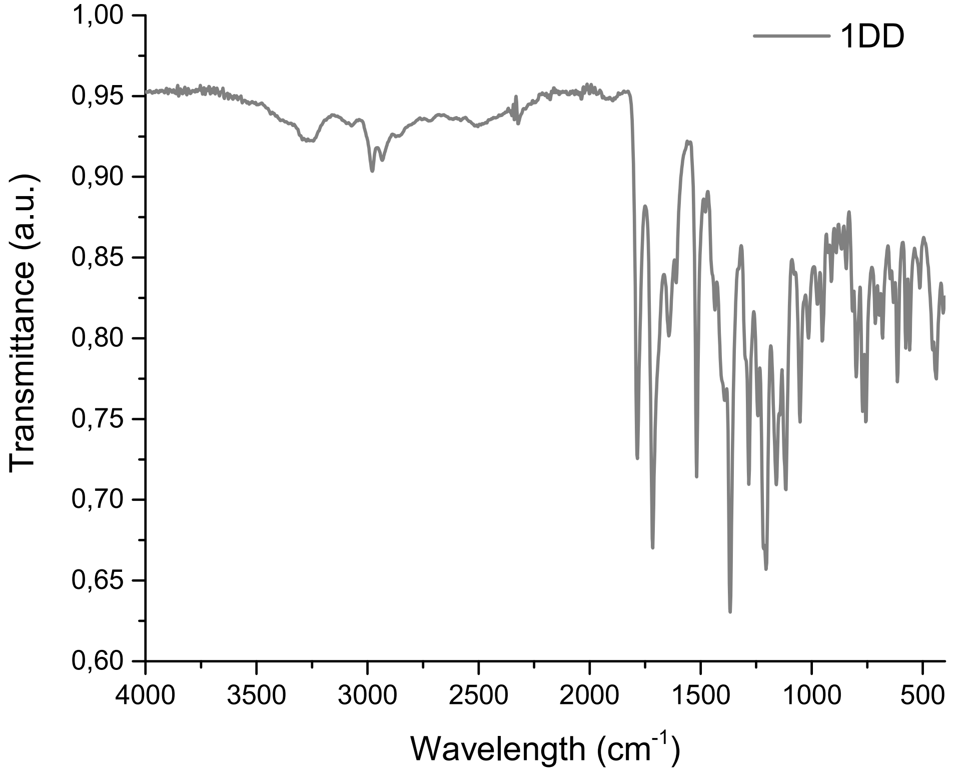

^1H NMR spectrum of Boc-D-F$_2$-Phe-L-Oxd-OH **C** in CD_3OD

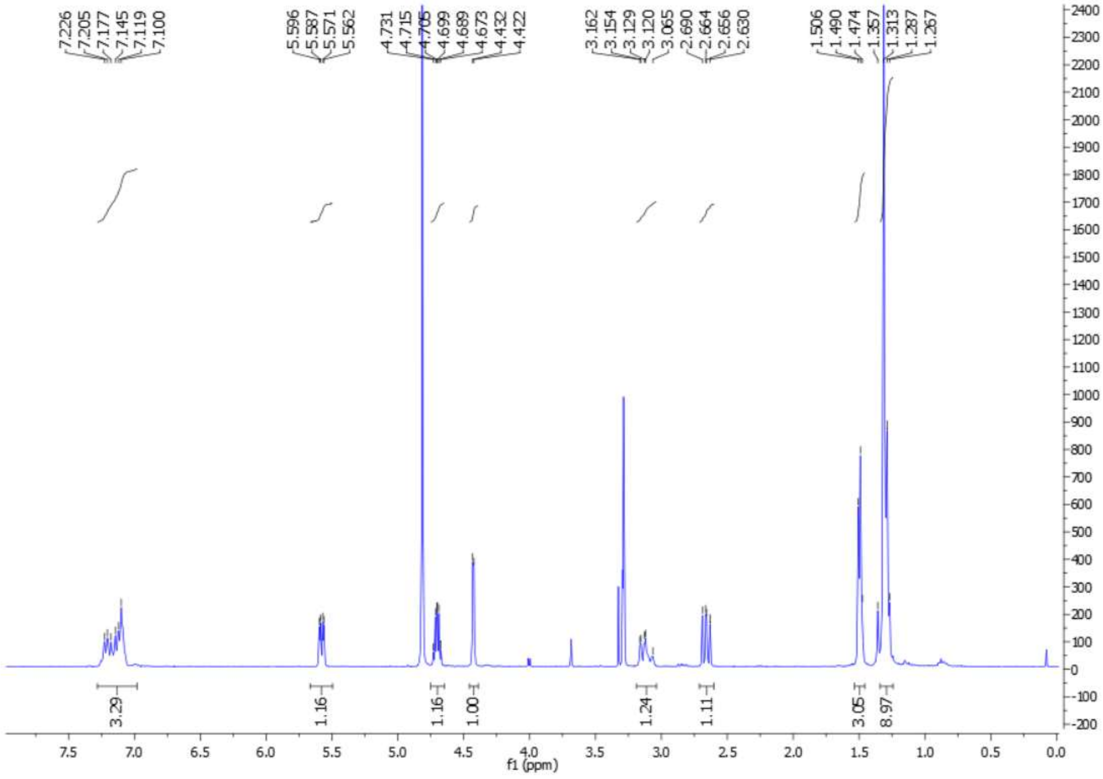

COSY spectrum of Boc-D-F$_2$-Phe-L-Oxd-OH **C** in $CDCl_3$

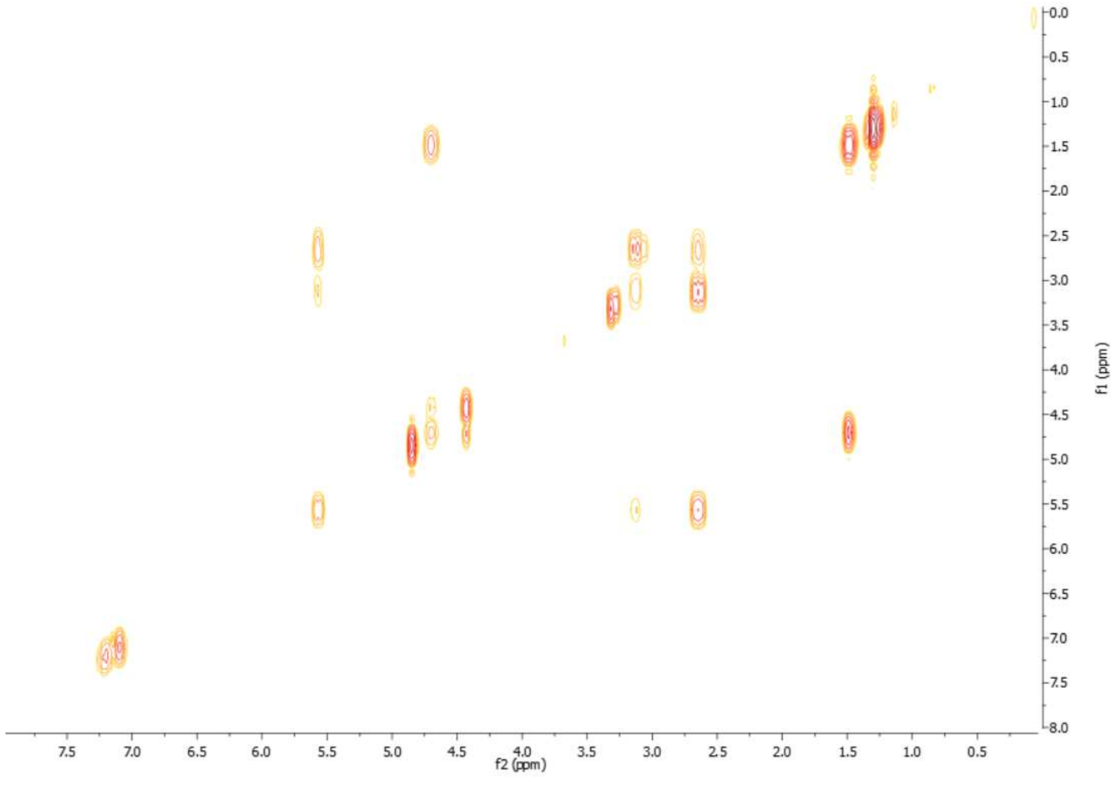

^{13}C NMR spectrum of Boc-D-F2-Phe-L-Oxd-OH **C** in CD_3OD

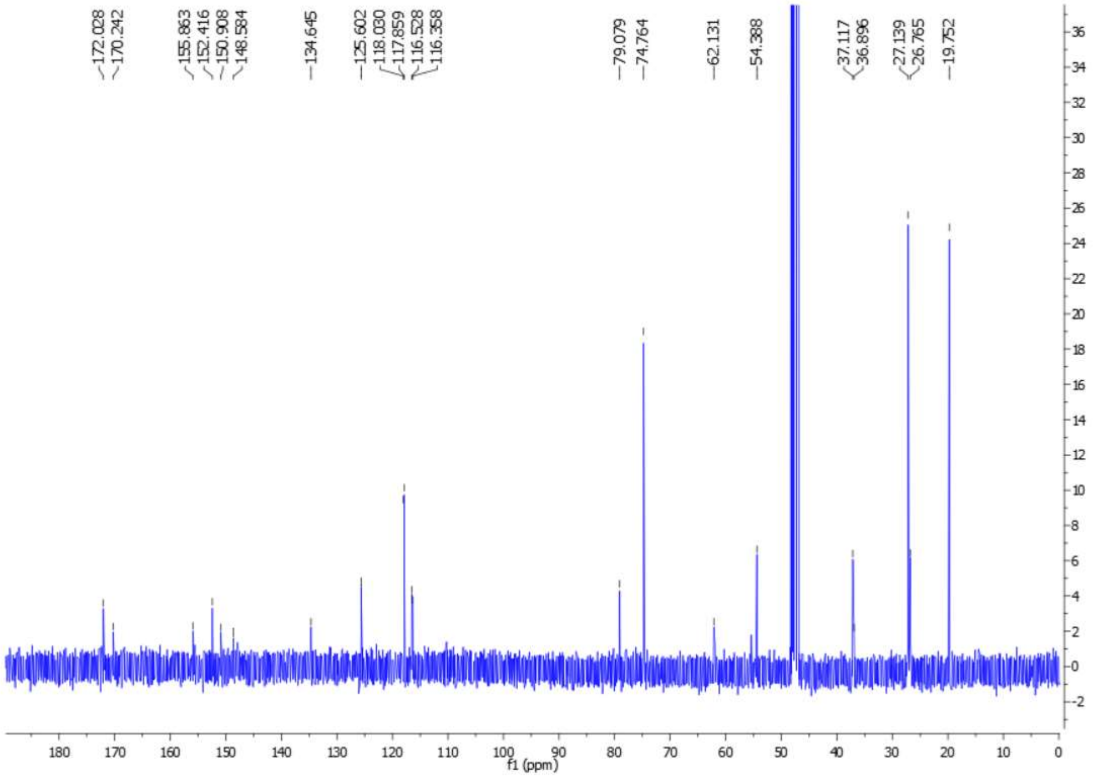

^{19}F NMR spectrum of Boc-D-F2-Phe-L-Oxd-OH **C** in $CDCl_3$

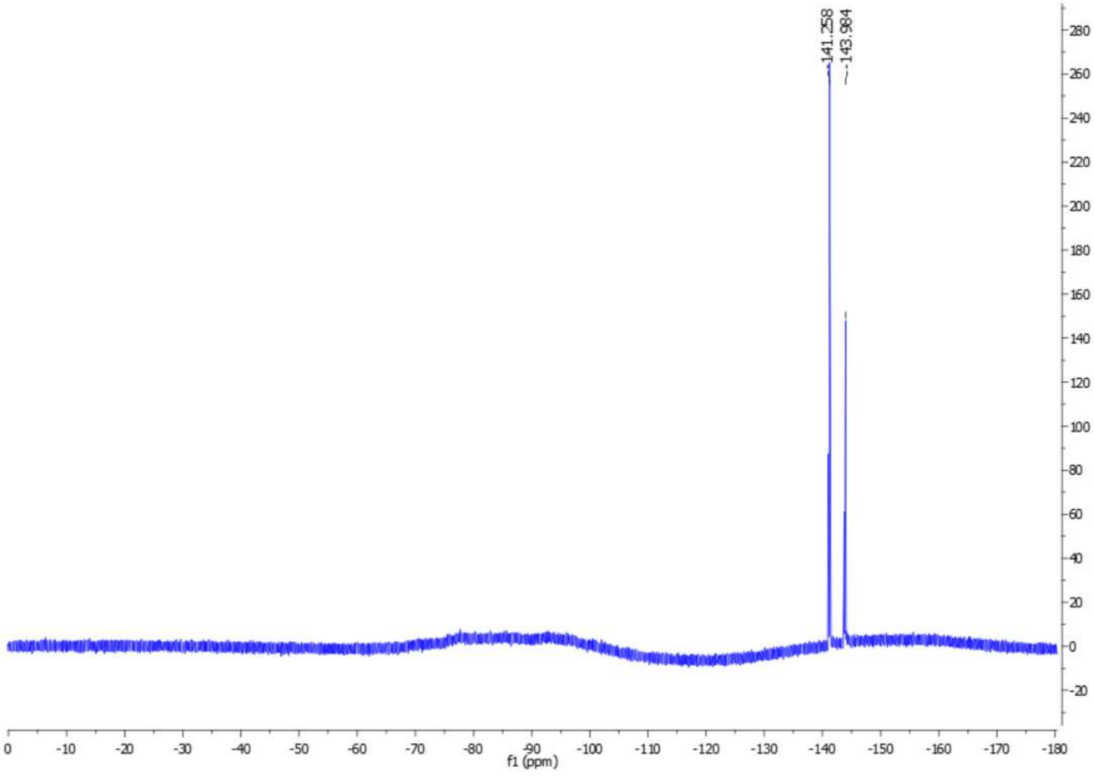

IR-ATR spectrum of Boc-D-F_2-Phe-L-Oxd-OH **C**

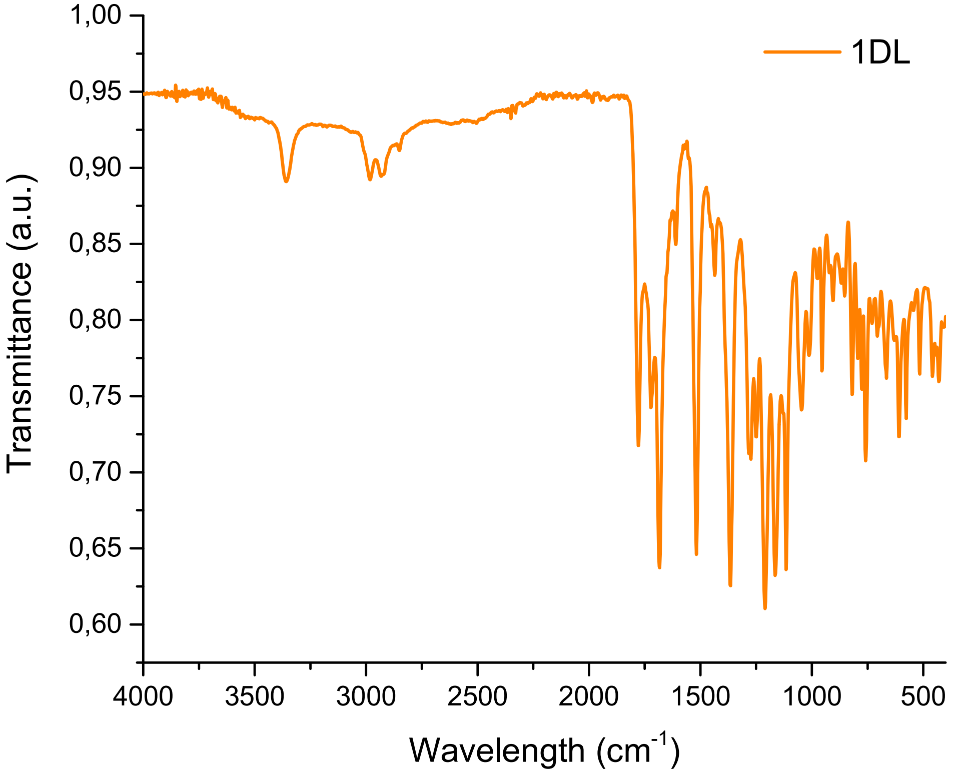

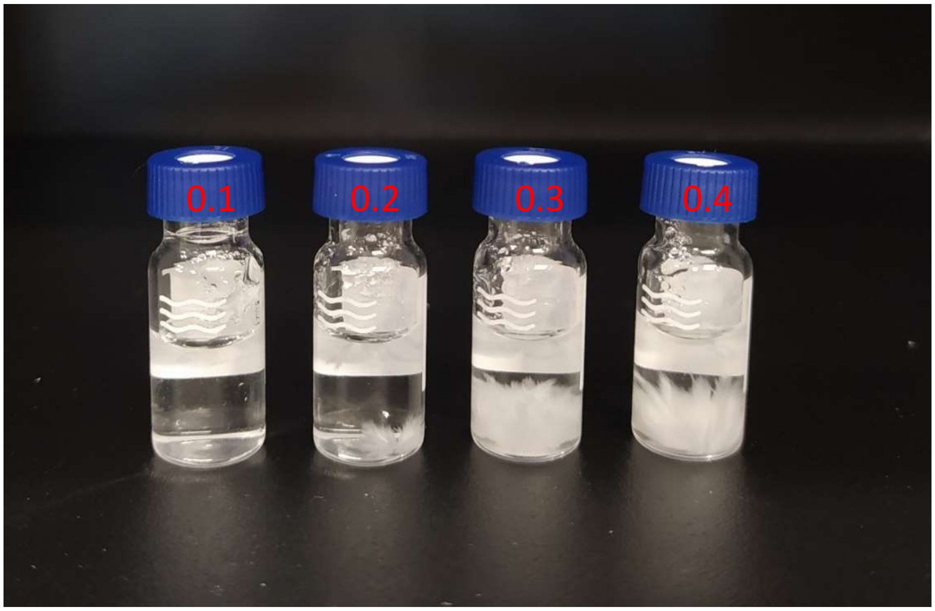

Figure S1. Analysis of the minimum gelation concentration (MGC) need to form hydrogels from **A**: from left to right: 0.1% w/w concentration; 0.2% w/w concentration; 0.3% w/w concentration; 0.4% w/w concentration.

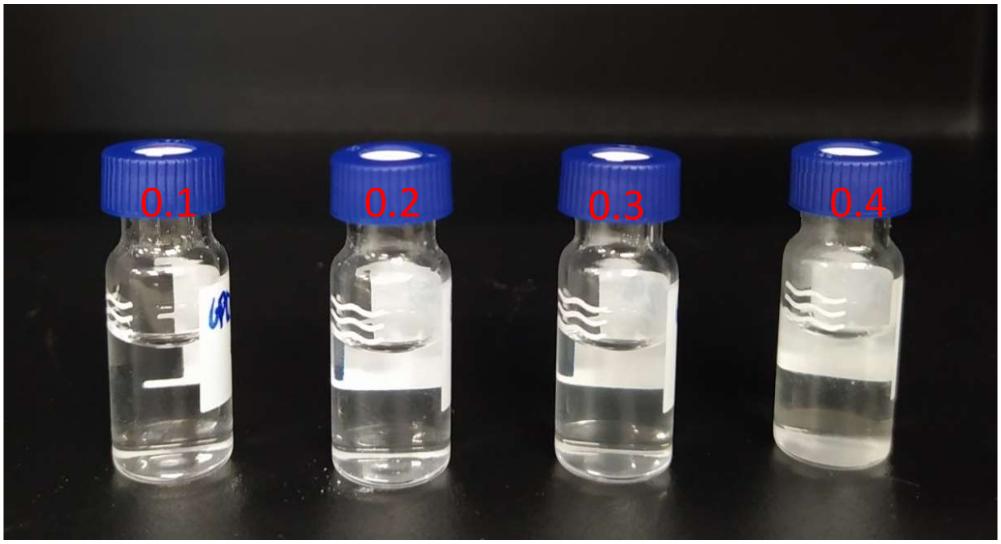

Figure S2. Analysis of the minimum gelation concentration (MGC) need to form hydrogels from **C**: from left to right: 0.1% w/w concentration; 0.2% w/w concentration; 0.3% w/w concentration; 0.4% w/w concentration.

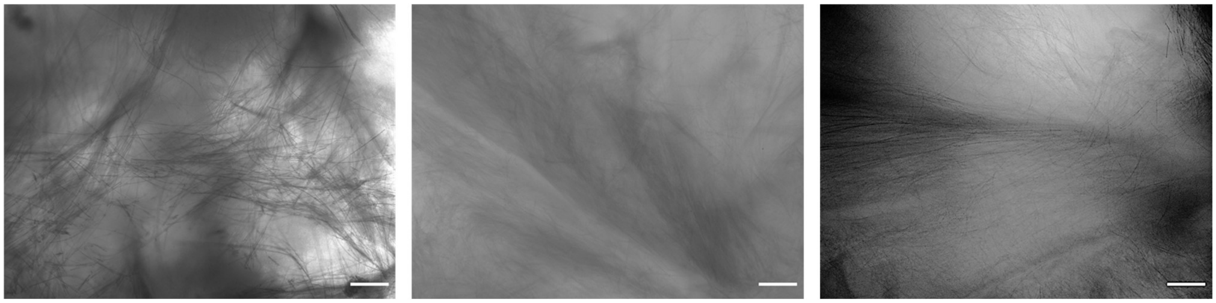

Figure S3. From left to right, hydrogel images of **1**, **2** and **3** obtained with an optic microscope with a 10x magnification. Scalebar: 100 μm.

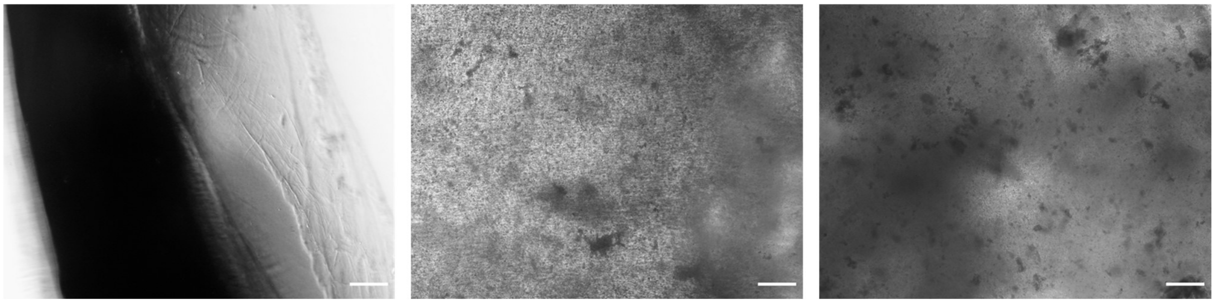

Figure S4. From left to right, hydrogel images of **7**, **8** and **9** obtained with an optic microscope with a 40x magnification. Scalebar: 25 μm.

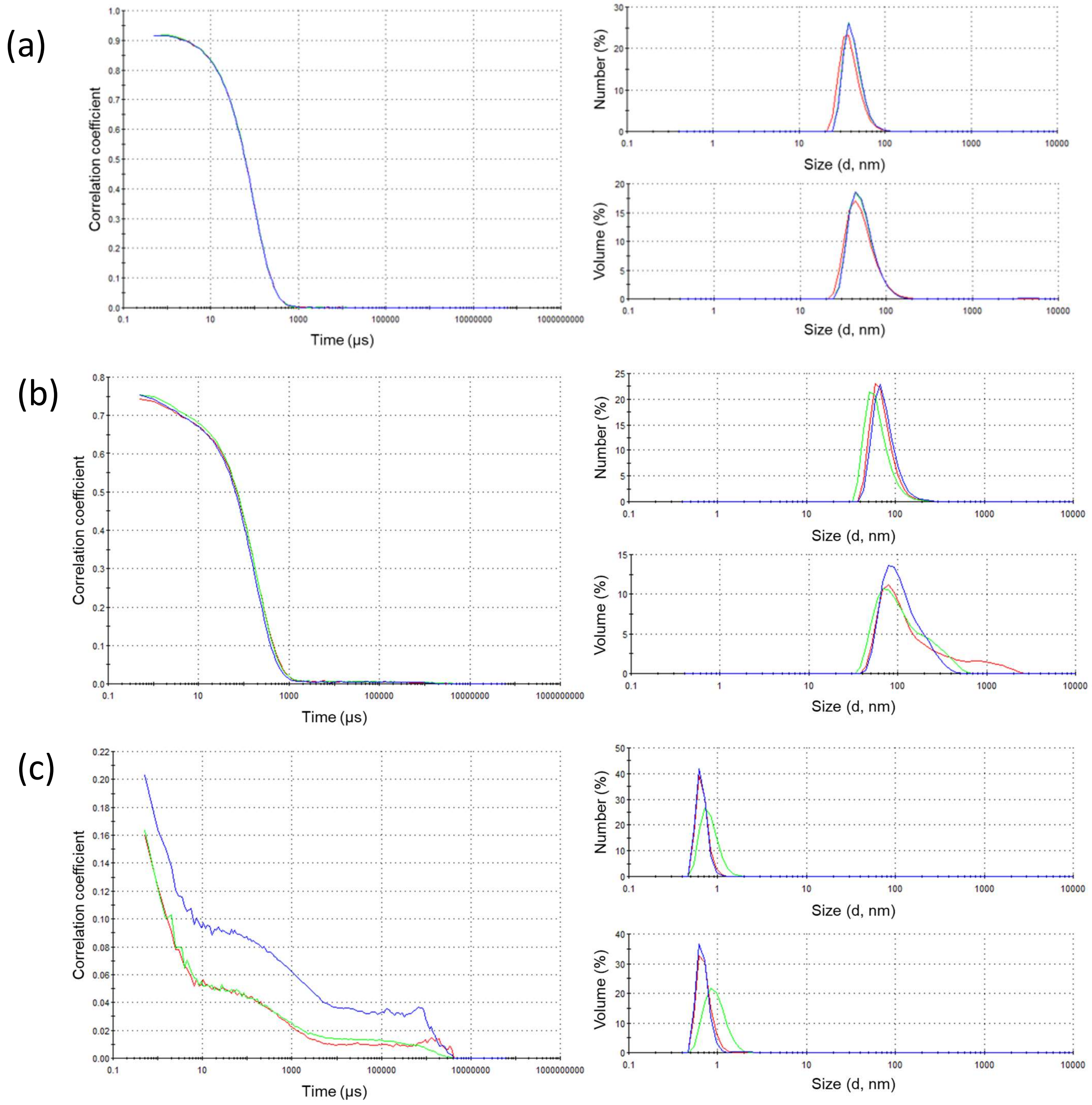

Figure S5. From top to bottom, DLS correlation coefficient, number and volume analysis of particles after filtration: (a) solution **4**; (b) solution **5**; (c) solution **6**.

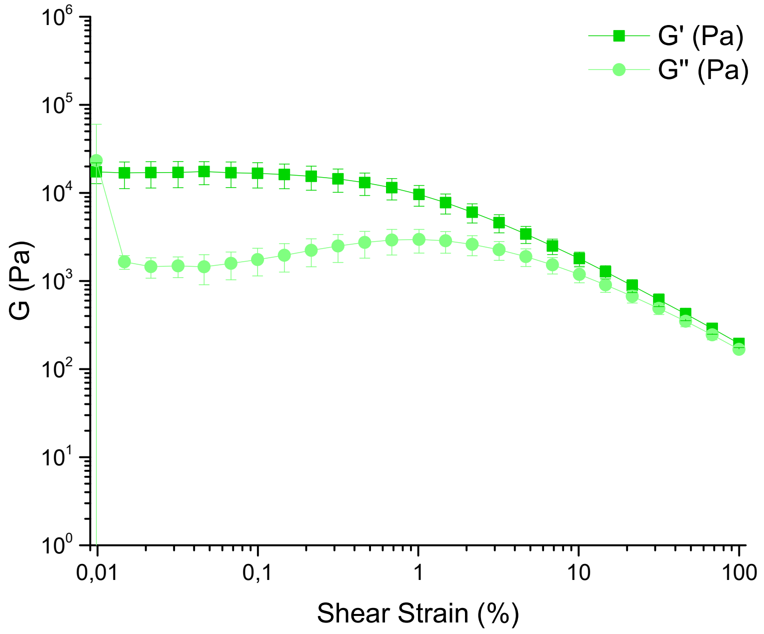

Figure S6. Amplitude sweep analysis of hydrogel **1**.

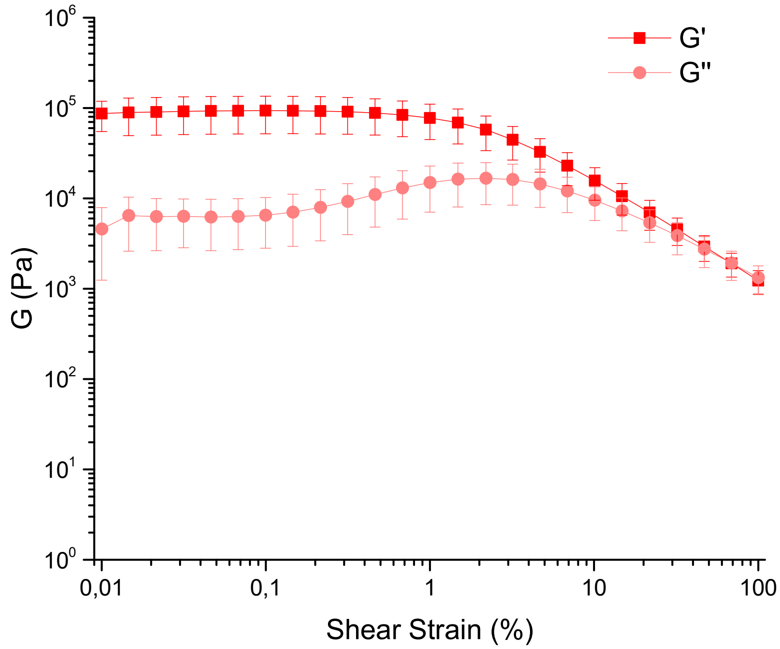

Figure S7. Amplitude sweep analysis of hydrogel **2**.

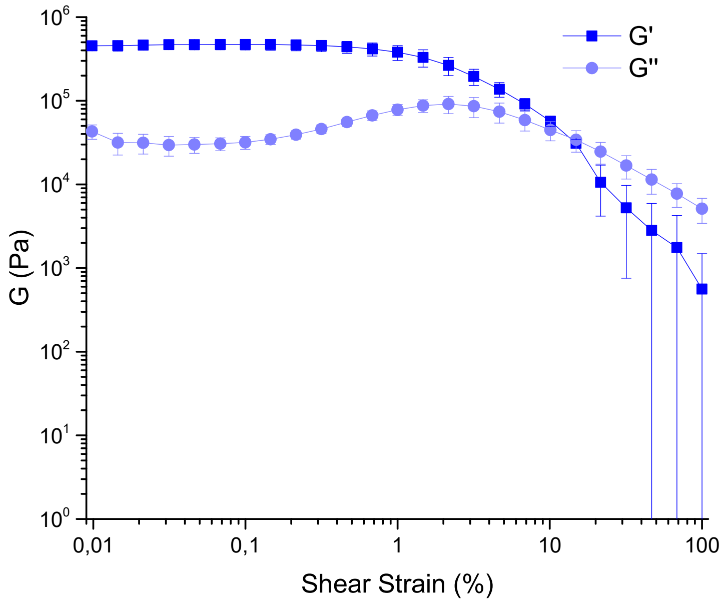

Figure S8. Amplitude sweep analysis of hydrogel **3**.

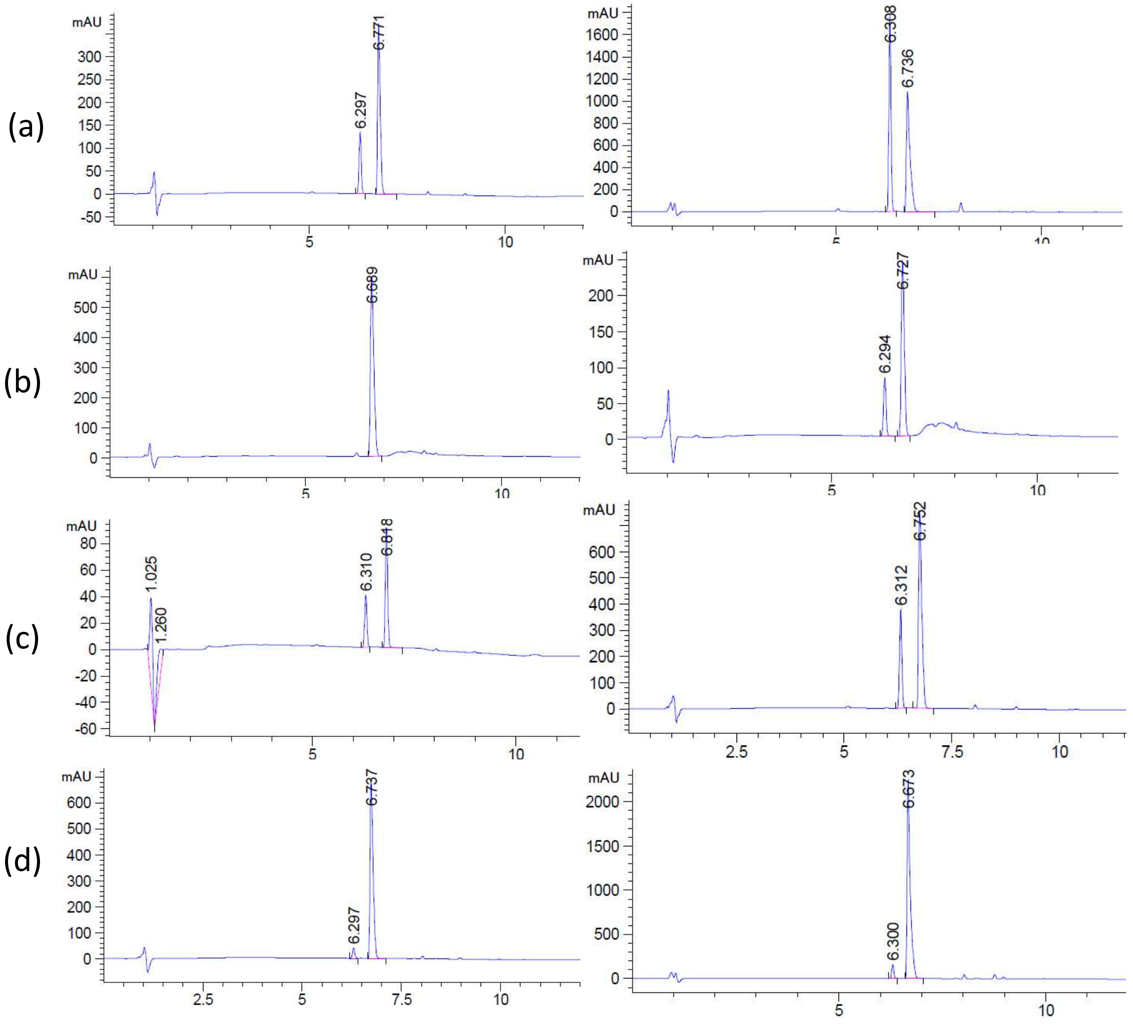

Figure S9. HPLC-MS analysis of gelators **B** and **C** before (left) and after (right) the addition of GdL: (a) gelator **B** (0.5 w/w concentration) in NaOH (no gel is formed); (b) gelator **B** (0.5% w/w concentration) in PBS (no gel is formed); (c) gelator **C** (0.5 w/w concentration) in NaOH (gel is formed); (d) gelator **C** (0.5% w/w concentration) in PBS (gel is formed). Retention times: gelator A (after hydrolysis) = 6.3 min; gelator B = 6.7 min; gelator C = 6.7 min.

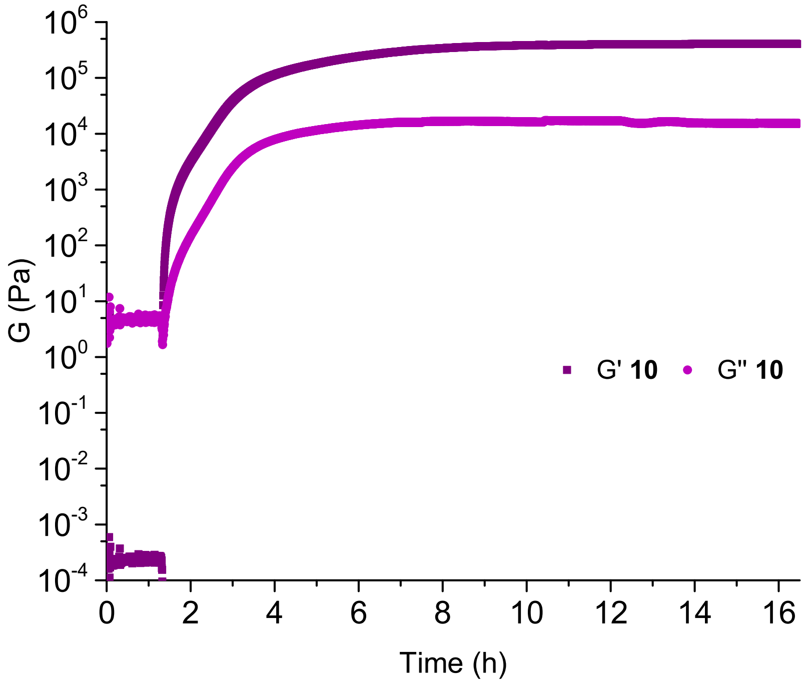

Figure S10. Time sweep analysis of hydrogel **10**.

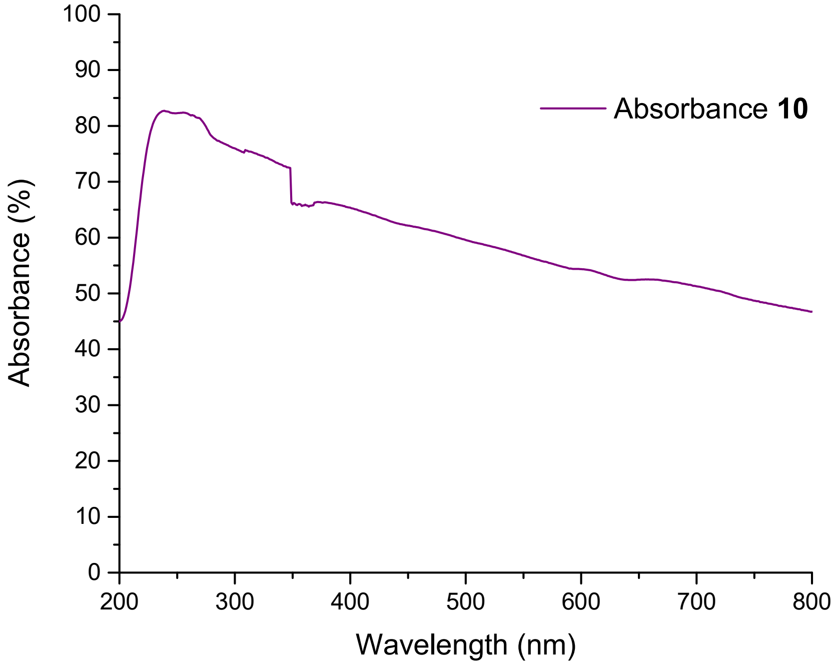

Figure S11. Absorbance spectrum of hydrogel **10**, collected using an optical path of 1.0 cm cuvette at 10 nm/s with a Cary300 UV-Vis double beam spectrophotometer, having a cuvette full of water as a reference.

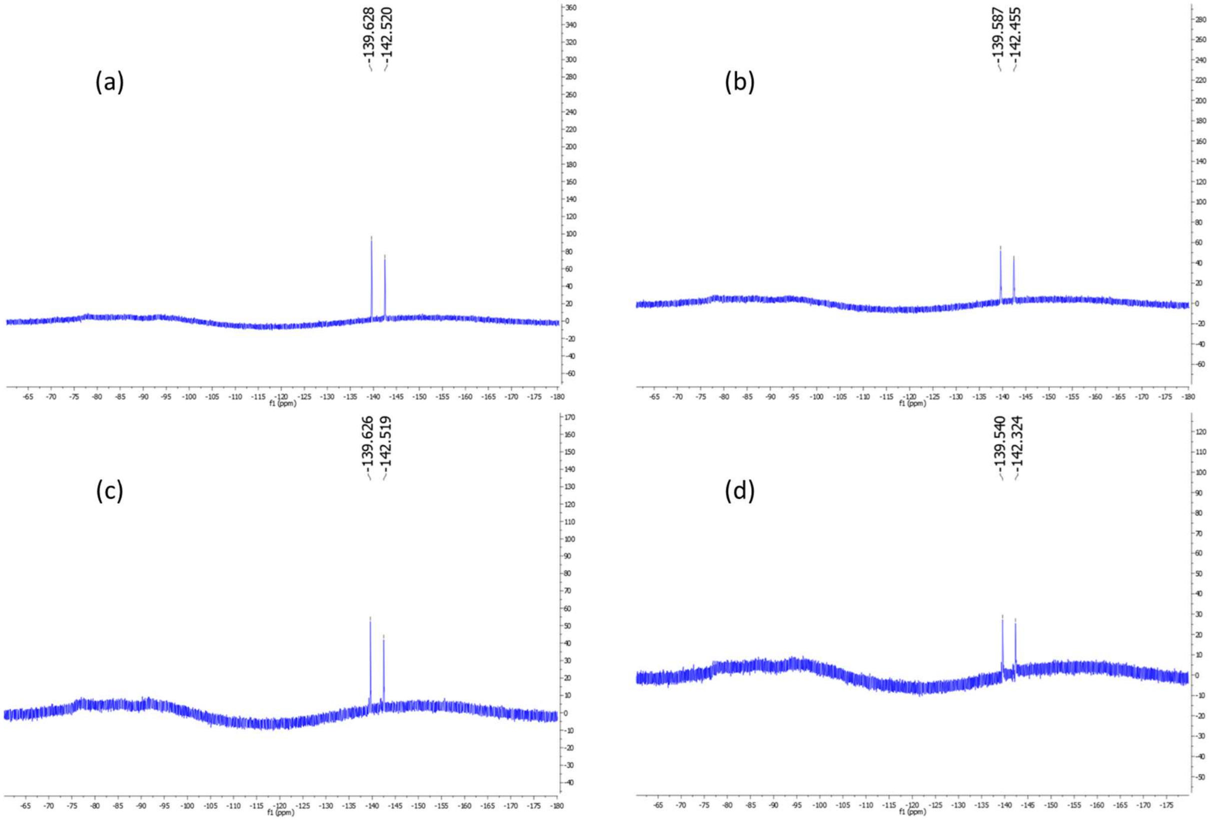

Figure S12. ^{19}F-NMR spectra registered in D_2O of gelator **A** (a) before the gelation process and (b) afterwards and gelator **C** (c) before the gelation process and (d) afterwards.
